# Supplementary material for: Rational Design of Resveratrol O-methyltransferase for the Production of Pinostilbene
Source: Int J Mol Sci. 2021 Apr 21;22(9):4345. doi: 10.3390/ijms22094345 (PMC8122247; doi:10.3390/ijms22094345)
Supplement: Supplementary file 1 [file ijms-22-04345-s001.zip › ijms-1180639 supplementary-revised.pdf]

Supplementary information

Rational design of resveratrol O-methyltransferase for the production of pinostilbene

Daniela P. Herrera [1], Andrea M. Chánique [1,2], Ascensión Martínez-Márquez [3], Roque Bru-Martínez [3], Robert Kourist [2], Loreto P. Parra [4,*] and Andreas Schüller [4,5,*]

[1] Department of Chemical and Bioprocesses Engineering, School of Engineering, Pontificia Universidad Católica de Chile, Vicuña Mackenna 4860, 7820244, Santiago, Chile; dpherrer@uc.cl (D.H.); amchanique@uc.cl (A.C.)

[2] Institute of Molecular Biotechnology, Graz University of Technology, Petersgasse 14, 8010 Graz, Austria; kourist@tugraz.at (R.K.)

[3] Department of Agrochemistry and Biochemistry, Faculty of Science and Multidisciplinary Institute for Environmental Studies "Ramon Margalef", University of Alicante, Alicante, Spain; roque.bru@gcloud.ua.es (R.B.M); asun.martinez@gcloud.ua.es (A.M.M)

[4] Institute for Biological and Medical Engineering, Schools of Engineering, Medicine and Biological Sciences, Pontificia Universidad Católica de Chile, Vicuña Mackenna 4860, 7820244, Santiago, Chile; (L.P.)

[5] Department of Molecular Genetics and Microbiology, School of Biological Sciences, Pontificia Universidad Católica de Chile, Av. libertador Bernardo O'Higgins 340, 8320000 Santiago, Chile; (A.S).

[*] To whom correspondence should be sent: aschueller@bio.puc.cl, lparraa@ing.puc.cl

Index

1 Supplementary Figures 3

Figure S1. Size exclusion chromatography (SEC) of W20A and F24A variants 3

Figure S2. Multiple sequence alignment of OMT-I with activity on stilbenes 4

Figure S3 Calibration curves for LC-MS/MS. 5

Figure S4. Example of LC-MS/MS transition chromatogram of stilbenes. 6

Figure S5. Purification of VvROMT Wt. and variants. 7

2 Supplementary Tables 8

Table S1. Specific and promiscuous OMT-I actives on stilbenes 8

Table S2. OMT-I used for the phylogenetic tree construction 9

Table S3. Primer sequences for variant construction by Gibson Assembly 11

Table S4. Primer sequence for VvROMT variants construction by QuickChange[a] 12

Table S5. LC-MS/MS parameters for targeted compounds 12

Table S6. Conversion rate of wildtype and VvROMT variants 13

3 Supplementary sequences 14

1 Supplementary Figures

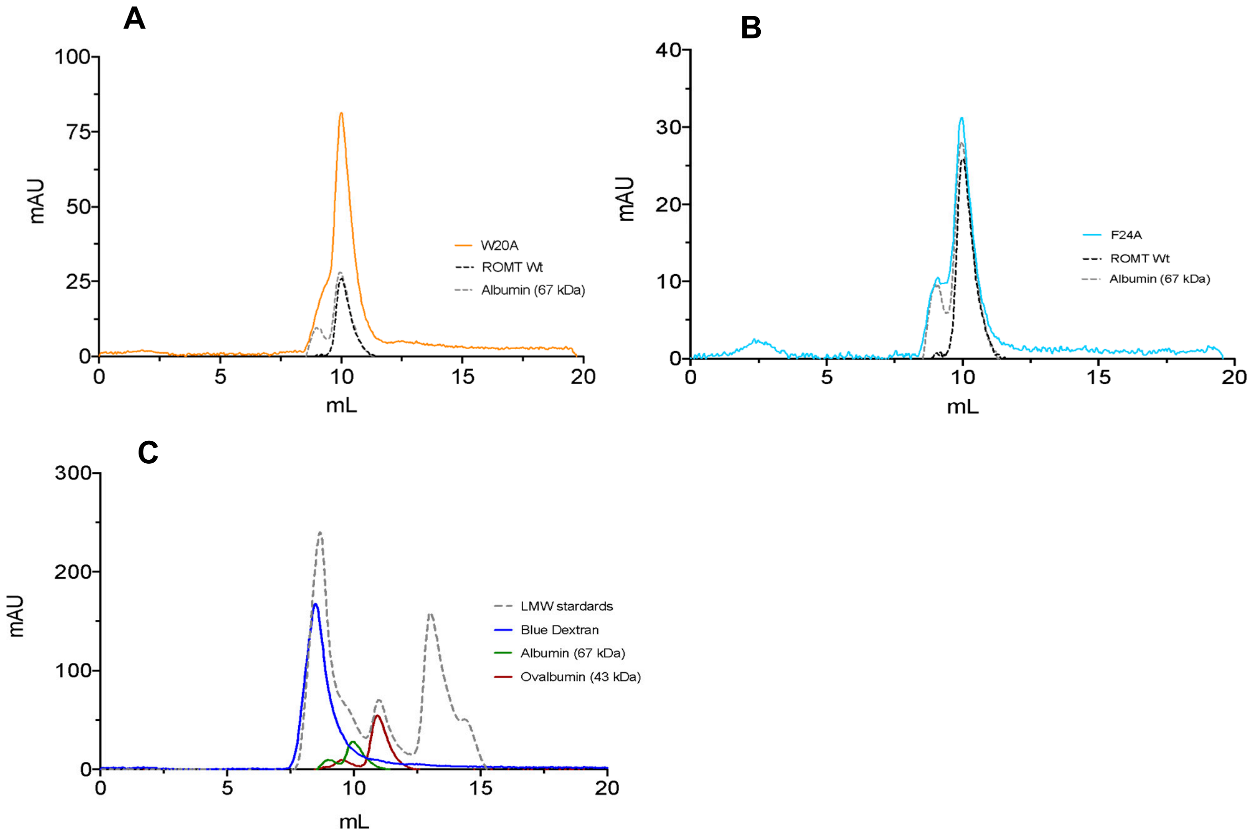

Figure S1. Size exclusion chromatography (SEC) of W20A and F24A variants.

Chromatograms of ROMT alanine variants W20A (Orange) (**A**), F24A (light blue) (**B**), and standard proteins (**C**). The absorbance at 280 nm against elution volume of the proteins is shown. The experiment was carried out using the SuperdeX 75 10/300 GL (GE Healthcare) column. Elution volume parameter (Kav) of W20A (0.078) (**A**) and F24A (0.079) (**B**) is comparable to the wildtype enzyme (0.079) black dashes lines, and to the Albumin standard protein (0.080), grey dashes lines, which is an indicator of the kDa that represent the VvROMT dimeric conformational state.

In **C**, the grey curve corresponds to a mix of the Low Molecular Weight standard (LMW) at 3µg/µL Blue dextran 2000, Albumin (67 kDa), Ovalbumin (45 kDa), Chymotrypsinogen A (25 kDa), and ribonuclease A (18.7 kDa). As a separate run, Blue dextran in blue, Albumin (67 kDa, Kav=0.0802) in green, and Ovalbumin (45 kDa, KaV=0.136) in red. All proteins were prepared at 2µg/µL, and a range of 150-250 µL of each one was used.

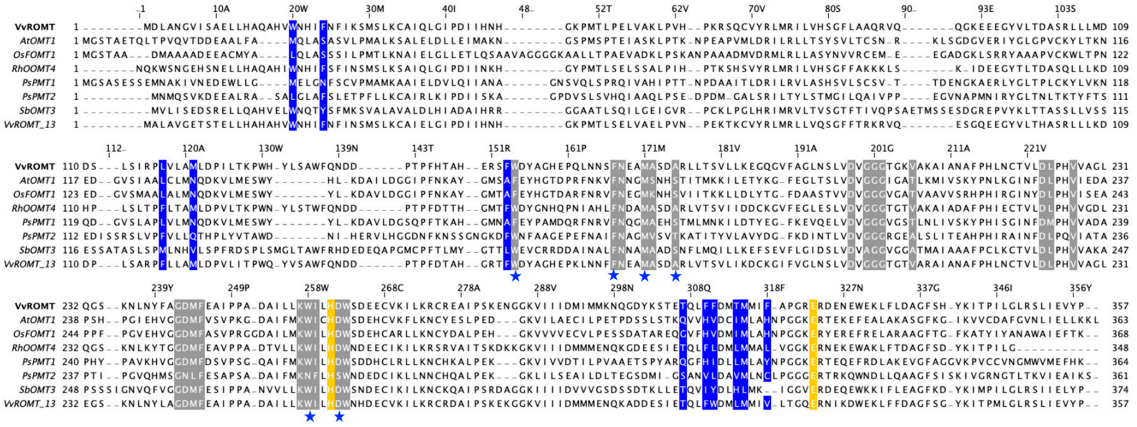

Figure S2. Multiple sequence alignment of OMT-I with activity on stilbenes

Substrate interaction residues are blue labeled, residues in contact with SAM are shown in grey. The catalytic residues are highlighted in yellow. The blue stars indicate residues that are in contact with both SAM and the substrate. The residue numbers over the alignment correspond to the VvROMT sequence.

Enzymes with 3/5-OH regioselectivity include VvROMT from *Vitis vinifera* (B6VJS4), RhOOMT4 from *Rosa hybrida* (Q8GU21), AtOMT1 from *Arabidopsis thaliana* (Q9FK25), and OsFOMT1 from *Oryza sativa* (Q6ZD89).

Enzymes with 3-OH regioselectivity correspond to PsPMT1, PsPMT2, from *Pinus sylvestris* (accession number AQX17823 and AQX17825). The enzymes SbOMT3, from *Sorghum bicolor* (A8QW52) and VvROMT13 preferentially, mono-methylate stilbenes.

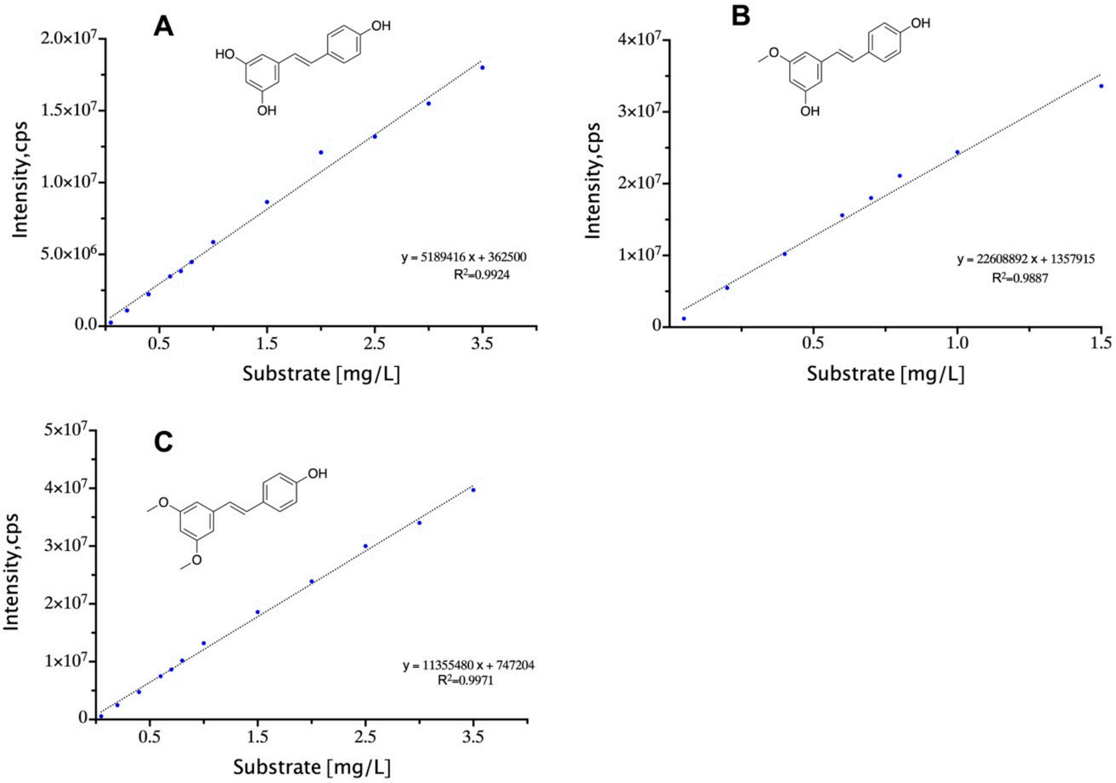

Figure S3 Calibration curves for LC-MS/MS.

The calibration curve for LC-MS/MS with the respective standard resveratrol (A) pinostilbene (B) and pterostilbene (C) was carried out in a concentration range between 0.05 mg L^{-1} to 3.5 mg L^{-1} with 10μL of injection.

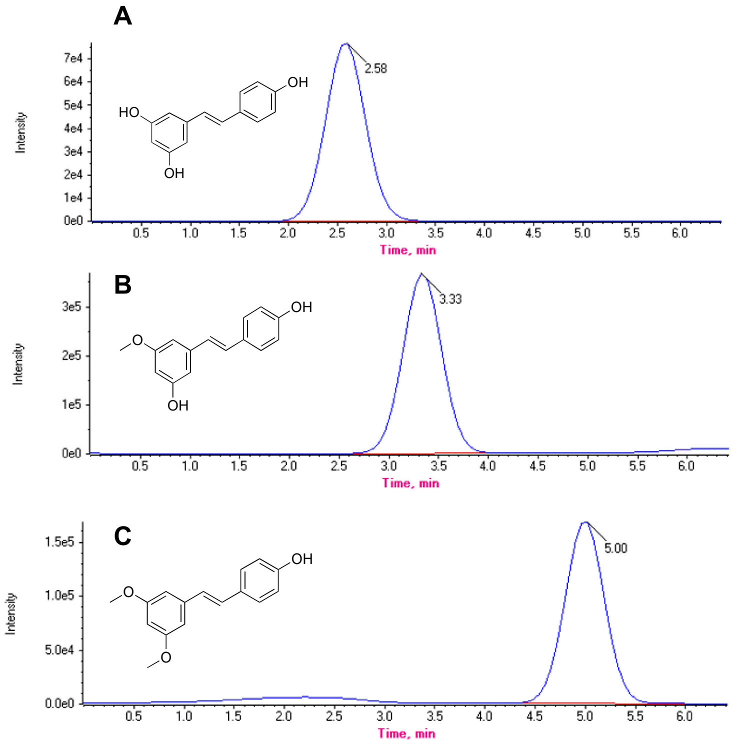

Figure S4. Example of LC-MS/MS transition chromatogram of stilbenes.

Resveratrol (**A**), pinostilbene (**B**) and pterostilbene (**C**) at 2.58, 3.33 and 5.00 of retention time and at 0.4 mg L^{-1}.

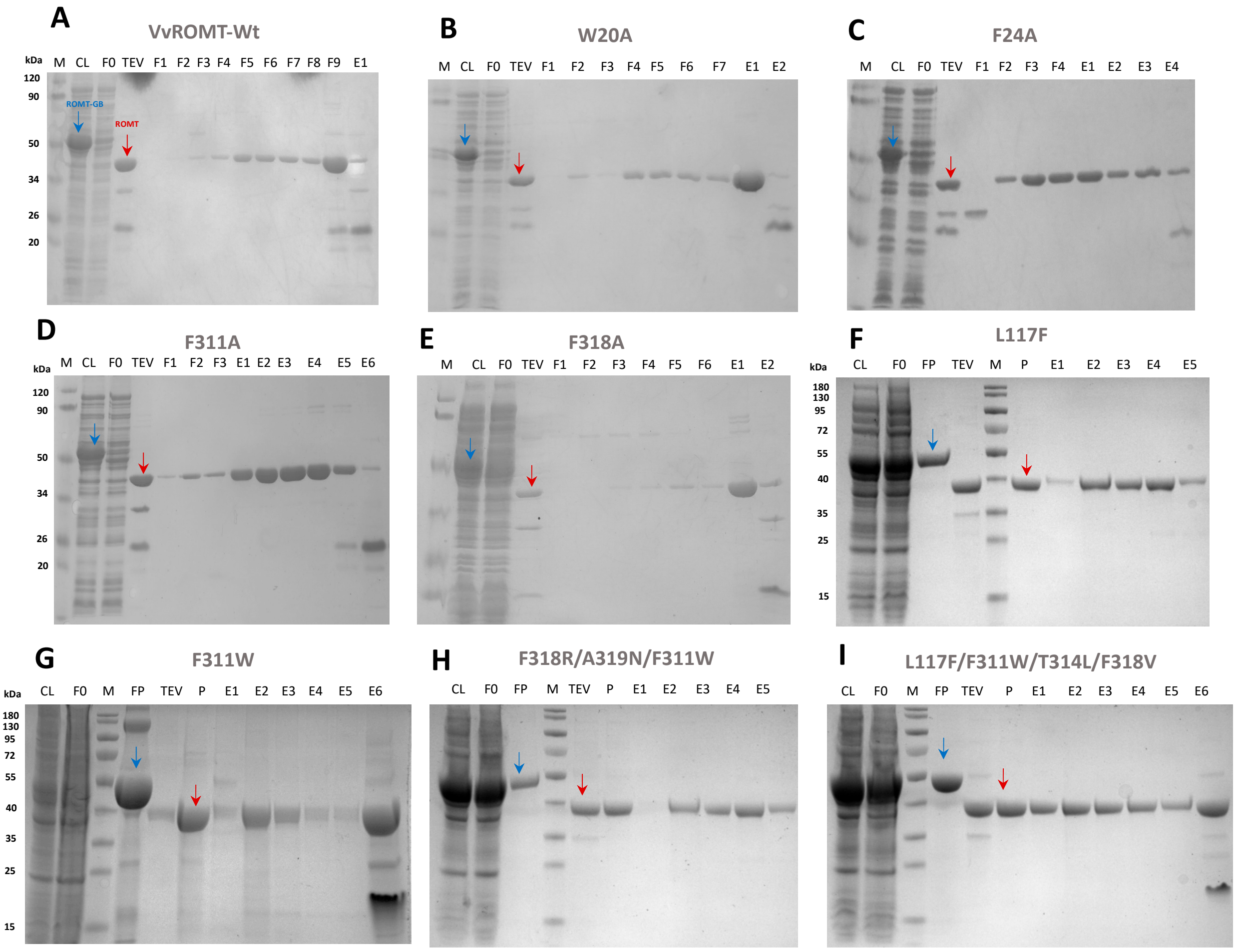

Figure S5. Purification of VvROMT Wt. and variants.

SDS-PAGE of purification of VvROMT Wildtype **(A)**, W20A **(B)**, F24A **(C)**, F311A **(D)**, F318A **(E)**, L117F **(F)**, F311W **(G)**, F318R/A319N/F311W **(H)** and L117F/F311W/T314L/F318L **(I)**. Lane M: protein molecular weight marker, CL: Cell Lysate, F0: Flowthrough after the first purification step, TEV: TEV cleavage, F1-F9: Flowthrough after the second purification, E1-E6: Elution of the second purification, FP: Fusion Protein and P: Purified protein. Blue arrows indicate VvROMT as a fusion protein (ROMT-GB, 47.8 kDa), and red arrows indicate VvROMT free after TEV cleave (~40.3 kDa).

2 Supplementary Tables

Table S1. Specific and promiscuous OMT-I actives on stilbenes.

Species	Enzyme name	Uniprot code	Substrate specificity	Stilbene regioselectivity	% Id seq. with ROMT	Reference [c]
Vitis vinifera	VvROMT	B6VJS4	Pinostilbene > Resveratrol	3/5-OH	100	Schmidlin et al., (2008) [28]
Arabidopsis thaliana	AtOMT1	Q9FK25	Flavone > Caffeic acid > Resveratrol > Pinostilbene	3/5-OH	33.2	Heo et al.,(2017) [26]
Oryza sativa	OsOMT1	Q6ZD89 [a]	Flavone > Pinosylvin> Resveratrol	3/5-OH	31.5	Kim et al., (2006) [33]
Rosa hybrida	RhOOMT4	Q8GU21	Orcinol > Resveratrol	3/5-OH	68.9	Martinez-Márquez et al., (2018) [31]
Pinus sylvestris	PsPMT2	AQX17823	Pinosylvin> Resveratrol	3-OH	30.7	Paasela et al., (2017) [35]
Pinus sylvestris	PsPMT1	AQX17825	Piceatannol > Caffeic acid > Flavonoid > Pinosylvin > Resveratrol	3-OH	31.4	Chiron et al., (2000) [34] Paasela et al., (2017) [35]
Sorghum bicolor	SbOMT3	A8QW53	Pentylresorcinol > Resorcinol > Resveratrol	3-OH [b]	45.9	Rimando et al., (2012) [27]
Sorghum bicolor	SbOMT1	A8QW52	Eugenol> Orcinol monomethyl ether > Resveratrol	4-OH	31.6	Rimando et al., (2012) [27]
Acorus calamus	AcOMT1	A0A5A4PX L6	Resveratrol > Isoraponthigenin	4-OH	51.6	Koeduka et al., (2018) [32]

[a] Descrived as pinosylvin methyltransferase by Kasuyana et al., (2007).
[b] Regioselectivity of 3 and 5 OH when is expressed in planta, but mostly 3-OH in bacteria.
[c] Reference number correspond to the principal manuscript.

Table S2. OMT-I used for the phylogenetic tree construction

Abbreviated enzyme name	UniProt code	Plant species	Phenylpropanoid group
VvROMT	B6VJS4	*Vitis vinifera*	Stilbenes
HiOMT3	B0ZB57	*Humulus lupulus*	n.c [1]
Mp8FOMT	Q6VMW0	*Mentha x piperita*	Flavonoids
CrFOMT	Q8GSN1	*Catharanthus roseus*	Flavonoids
ObCVOMT1	Q93WU3	*Ocimum basilicum*	Phenylpropenes
ObEOMT1	Q93WU2	*Ocimum basilicum*	Phenylpropenes
Ge7IOMT	Q84KK5	*Glycyrrhiza echinata*	Isoflavonoids
Ms7IOMT9	O22309	*Medicago sativa*	Isoflavonoids
Ms7IOMT6	O22308	*Medicago sativa*	Isoflavonoids
Lj4IOMT	Q84KK4	*Lotus japonicus*	Isoflavonoids
Mt4IOMT	Q29U70	*Medicago truncatula*	Isoflavonoids
Gm4FOMT2	C6TAY1	*Glycine max*	Isoflavonoids
ZmOMT	P47917	*Zea mays*	Alkylresorcinol
Cj6OMT	Q9LEL6	*Coptis japonica*	Alkaloids
Ig4OMT	Q84KK6	*Glycyrrhiza echinata*	Isoflavonoids
SbOMT3	A8QW53	*Sorghum bicolor*	Alkylresorcinol
Ps4OMT2	Q7XB10	*Papaver somniferum*	Alkaloids
Cj4OMT	Q9LEL5	*Coptis japonica*	Alkaloids
OsFOMT	Q53QK0	*Oryza sativa*	Flavonoids
Ps6OMT	Q6WUC1	*Papaver somniferum*	Alkaloids
Ps4OMT1	Q7XB11	*Papaver somniferum*	Alkaloids
Ps7OMT	Q6WUC2	*Papaver somniferum*	Alkaloids
PsN7OMT	C7SDN9	*Papaver somniferum*	Alkaloids
HmOMT2	B0ZB56	*Humulus lupulus*	Chalcones
CjOMT	Q8H9A8	*Coptis japonica*	Alkaloids
HlOMT1	B0ZB55	*Humulus lupulus*	Chalcones
TaFOMT2	Q38J50	*Triticum aestivum*	Flavonoids
SoCOMT1	O82054	*Saccharum officinarum*	Phenylpropenes
CaCOMT1	Q9FQY8	*Capsicum annuum*	Phenylpropenes
AtOMT1	Q9FK25	*Arabidopsis thaliana*	Flavonoids
PtCOMT1	Q00763	*Populus tremuloides*	Phenylpropenes

CaFOMT2	Q42653	*Chrysosplenium americanum*	Flavonoids
CaFOMT1	P59049	*Chrysosplenium americanum*	Flavonoids
PtCOMT2	Q41086	*Populus tremuloides*	Phenylpropenes
PsPgCOMT1	Q43046	*P. sieboldii x P. grandidentata*	Phenylpropenes
CbCOMT1	O23760	*Clarkia breweri*	Phenylpropenes
PsPgCOMT3	Q43047	*P. sieboldii x P. grandidentata*	Phenylpropenes
CcCOMT	O81646	*Capsicum chinense*	Phenylpropenes
CcCOMT1	Q8LL87	*Coffea canephora*	Phenylpropenes
ZmCOMT1	Q06509	*Zea mays*	Phenylpropenes
RsCOMT1	Q8GU25	*Rosa chinensis*	Phenylpropenes
ObCOMT1	Q9XGW0	*Ocimum basilicum*	Phenylpropenes
CrCOMT1	Q8W013	*Catharanthus roseus*	Phenylpropenes
TaFOMT1	Q84N28	*Triticum aestivum*	Flavonoids
PdCOMT1	Q43609	*Prunus dulcis*	Phenylpropenes
EguCOMT1	P46484	*Eucalyptus gunnii*	Phenylpropenes
AmCOMT1	Q6T1F5	*Ammi majus*	Phenylpropenes
ObCOMT2	Q9XGV9	*Ocimum basilicum*	Phenylpropenes
EglCOMT1	Q9SWC2	*Eucalyptus globulus*	Phenylpropenes
SbOMT1	A8QW52	*Sorghum bicolor*	Phenylpropenes
Os7FOMT	Q0IP69	*Oryza sativa*	Flavonoids
PaAMT1	B8RCD3	*Pimpinella anisum*	Phenylpropenes
OsFOMT1	Q6ZD89	*Oryza sativa Japonica Group*	Flavonoids
PsPMT2	AQX17823	*Pinus sylvestris*	Stilbenes
PsPMT1	AQX17825	*Pinus sylvestris*	Flavonoids
VrROMT	K7XQ68	*Vitis riparia*	Stilbenes
RhOOMT1	Q8L5K8	*Rosa hybrid cultivar*	Phenylpropenes
RhOOMT2	Q8L5K7	*Rosa hybrid cultivar*	Phenylpropenes
RhOOMT4	Q8GU21	*Rosa hybrid cultivar*	Phenylpropenes
AcOMT1	A0A5A4	*Acorus calamus*	Stilbenes
PlOMT	V9W3E0	*Paenibacillus larvae*	Outgroup
CsOMT	B0EXJ8	*Catharanthus roseus*	Alkaloids
Ps3IOMT1	O24305	*Pisum sativum*	Isoflavonoids
Ps3IOMT2	P0DH60	*Pisum sativum*	Isoflavonoids
Ms7IOMT8	O24529	*Medicago sativa*	Isoflavonoids
MtIOMT3	Q06YR3	*Medicago truncatula*	Isoflavonoids
LpCOMT1	Q9ZTU2	*Lolium perenne*	Phenylpropenes
MsCOMT1	P28002	*Medicago sativa*	Phenylpropenes
Tf6OMT	Q5C9L7	*Thalictrum flavum*	Alkaloids

CbIEMT1	O04385	*Clarkia breweri*	Phenylpropenes

[1] n.c. Not classified

Table S3. Primer sequences for variant construction by Gibson Assembly

VvROMT variant [1]	Forward 5' to 3'	Reverse 5' to 3'	Fragment Size (pb)	Template
W20A_1	CGTT**gcg**AACCACATTTTCAACTTTATC	CTGAAACATGGCAAAGGTAGCGT	2,401	Wt
W20A_2	CAACGCTACCTTTGCCATGTTTCAG	GATAAAGTTGAAAATGTGGTT**cgc**AACGTG	4,156	Wt
F24A_1	CCACATT**gcg**AACTTTATCAAGAGCATGAG	CTGAAACATGGCAAAGGTAGCGT	2,391	Wt
F24A_2	CAACGCTACCTTTGCCATGTTTCAG	CTCATGCTCTTGATAAAGTT**cgc**AATGTGG	4,166	Wt
F311A-1	ACTGTTC**gcg**GACATGACCATGAT	CGGATGCCGGGAGCAGACAA	2,788	Wt
F311A_2	TTGTCTGCTCCCGGCATCCG	ATCATGGTCATGTC**cgc**GAACAGT	3,769	Wt
F318A_1	ATGACCATGATGATC**gcg**GCGC	CGGATGCCGGGAGCAGACAA	2,775	Wt
F318A_2	TTGTCTGCTCCCGGCATCCG	GCGC**cgc**GATCATCATGGTCAT	3,782	Wt
L117F-1	AGCATTCGTCCG**ttc**GTGCTGG	CGGATGCCGGGAGCAGACAA	3375	Wt
L117F-2	TTGTCTGCTCCCGGCATCCG	CCAGCAC**gaa**CGGACGAATGCT	3182	Wt
L117F/F318L-1	ATGATGATC**ctg**GCGCCGGGT	CGGATGCCGGGAGCAGACAA	2775	L117F
L117F/F318L-2	TTGTCTGCTCCCGGCATCCG	ACCCGGCGC**cag**GATCATCAT	3782	L117F

[1] Alanine variants were generated using pET25GB1_ROMT vector as a template (wild type). For the variant L117F/318, it was used pET25GB1_L117F as a template. Bold lowercase letters indicate the respective codon modification.

Table S4. Primer sequence for VvROMT variants construction by QuickChange [1]

ROMT Variant	Forward 5' to 3'	Reverse 5' to 3'	Template
F311W	ACCGAGACGCAACTGTTCT**gg**GACATGACCATGATGATC	GATCATCATGGTCATGT**cc**CAGAACAGTTGCGTCTCGGT	WT
L117F/F311W	ACCGAGACGCAACTGTTCT**gg**GACATGACCATGATGATC	GATCATCATGGTCATGT**cc**CAGAACAGTTGCGTCTCGGT	L117F
L117F/F311W T314L/F318V	CAACTGTTCTGGGACATG**cta**ATGATGATC**g**TCGCGCCGGGTCGTGAAC	GTTCACGACCCGGCGCGA**c**GATC**atc**ATTAGCATGTCCCAGAACAGTTG	L117F/311W
F318R/A319N	CAACTGTTCTTTGACATGACCATGATGATC**cg**C**aat**CCGGGTCGTGAACGTGACG	CGTCACGTTCACGACCCGG**att**G**cg**GATCATCATGGTCATGTCAAAGAACAGTTG	Wt
F318Y/A319N	CGCAACTGTTCTTTGACATGACCATGATGATCT**ataat**CCGGGTCGTGAACGTGA	TCACGTTCACGACCCGG**attat**AGATCATCATGGTCATGTCAAAGAACAGTTGCG	Wt
F311W /F318R/A319N	ACCGAGACGCAACTGTTCT**gg**GACATGACCATGATGATC	GATCATCATGGTCATGT**cc**CAGAACAGTTGCGTCTCGGT	F318Y/A319N
L117F/F311L/T314L/F318L	CGGCGCCAGGATCATCAT**tag**CATGTC**taa**GAACAGTTGCGTCTCGGTG	CACCGAGACGCAACTGTTC**tta**GACATG**cta**ATGATGATCCTGGCGCCG	L117F/318L

[1] Variant name, primer sequence and the pET25GB1 plasmid used as a template are indicated. Bold lowercase letters correspond to codon modification.

Table S5. LC-MS/MS parameters for targeted compounds

Compound	t_R (min)[a]	MRM transition 1 (*m/z*)	DP (V)[b]	CE (eV)[c]	CXP (V)[d]	MRM transition 2 (*m/z*)	DP (V)	CE (eV)	CXP (V)
Resveratrol	2.53	227.000 > 143.000	-90.00	-34.00	-5.00	227.000 > 184.900	-90.0	-26.00	-15.00
Pinostilbene	3.31	241.034 > 224.900	-75.00	-30.00	-15.00	241.034 > 180.900	-75.0	-40.00	-11.00
Pterostilbene	4.95	255.148 > 239.900	-105.00	-24.00	-7.00	255.148 > 196.900	-105.0	-38.00	-15.00

[a] t_R: Retention time, [b] DP: Declustering potential, [c] CE: Collision energy, [d] CXP: Collision cell exit potential.

24h reaction	From Resveratrol					From Pinostilbene	
Enzyme	μM PIN	μM PTS	% Conversion to PIN	% Conversion to PTS	% Total Conversion	μM PTS	% Conversion to PTS
Wt	n.c [a]	128.7	n.c	44.2	44.2	239.1	88.7
F311W	39.7	94.3	13.3	31.5	44.7	98.2	30.0
L117F	168.5	71.0	49.6	20.9	70.5	240.0	89.7
L117/F311W	222.2	23.3	66.6	7.0	73.6	61.3	19.1
L117F/F311W/T314L/F318V	96.69	5.60	34.9	2.0	36.9	15.5	4.8
L117F/F311W/T314L/F318L	23.51	5.02	9.6	2.0	11.7	12.9	4.2
F318Y/A319N	<1	46.11	<1	17.5	17.5	60.1	19.1
F318R/A319N	16.07	n.c	7.2	0%	7.2	11.0	3.4
F318R/A319N/F311W	<1	n.c	<1	n.c	<1	n.c	n.c

Table S6. Conversion rate of wildtype and VvROMT variants

[a] n.c.: Not converted

3 Supplementary sequences

Sequence S1. Nucleotide synthesized sequence of VvROMT*.*

Accession no. FM178870. Codons were optimized for *E. coli* protein expression; bold letters correspond to the *NdeI* and *XhoI* restriction sites used for cloning.

>**CATATG**GACCTGGCGAACGGCGTGATTAGCGCGGAGCTGCTGCATGCGCAGGCGCACGTTTGGAACCACATTTTCAA
CTTTATCAAGAGCATGAGCCTGAAATGCGCGATTCAACTGGGCATCCCGGATATCATTCACAACCACGGCAAGCCGAT
GACCCTGCCGGAACTGGTGGCGAAGCTGCCGGTTCACCCGAAACGTAGCCAGTGCGTGTACCGTCTGATGCGTATCCT
GGTTCACAGCGGTTTTCTGGCGGCGCAACGTGTGCAGCAAGGCAAAGAGGAAGAGGGTTATGTTCTGACCGACGCGA
GCCGTCTGCTGCTGATGGACGATAGCCTGAGCATTCGTCCGCTGGTGCTGGCGATGCTGGATCCGATCCTGACCAAAC
CGTGGCACTACCTGAGCGCGTGGTTCCAGAACGACGATCCGACCCCGTTTCACACCGCGCACGAGCGTAGCTTCTGGG
ACTATGCGGGCCACGAGCCGCAACTGAACAACAGCTTTAACGAAGCGATGGCGAGCGATGCGCGTCTGCTGACCAGC
GTGCTGCTGAAAGAAGGCCAGGGTGTTTTCGCGGGCCTGAACAGCCTGGTGGACGTTGGTGGCGGTACCGGCAAGGT
GGCGAAAGCGATTGCGAACGCGTTTCCGCACCTGAACTGCACCGTTCTGGATCTGCCGCACGTGGTTGCGGGCCTGCA
AGGTAGCAAGAACCTGAACTACTTCGCGGGTGATATGTTTGAGGCGATCCCGCCGGCGGATGCGATTCTGCTGAAATG
GATCCTGCACGACTGGAGCGATGAAGAGTGCGTGAAGATTCTGAAACGTTGCCGTGAGGCGATCCCGAGCAAGGAAA
ACGGCGGTAAAGTTATCATCATCGACATGATCATGATGAAGAACCAGGGCGATTATAAAAGCACCGAGACGCAACTGT
TCTTTGACATGACCATGATGATCTTCGCGCCGGGTCGTGAACGTGACGAAAACGAGTGGGAAAAGCTGTTCCTGGATG
CGGGCTTTAGCCACTACAAAATTACCCCGATCCTGGGTCTGCGTAGCCTGATCGAAGTTTATCCGTAA**CTCGAG**
